# Multidisciplinary Approach in the Diagnosis and Treatment of Twin Anemia Polycythemia Sequence in Monochorionic Twin Pregnancies: Case Report

**DOI:** 10.3390/life14091071

**Published:** 2024-08-27

**Authors:** Marina Fortún Agud, María Marfil González, Susana Monis Rodríguez, Maria Suarez Arana, Marta Blasco Alonso, Jesus Jimenez Lopez, Isidoro Narbona Arias

**Affiliations:** 1 Obstetrics and Gynecology, Materno-Infantil Hospital Regional Universitaria Málaga, Avenue Arroyo de los Ángeles S/N, 29011 Málaga, Spain; mnfortun@gmail.com (M.F.A.); mariamarfil97@gmail.com (M.M.G.); monissusana@gmail.com (S.M.R.); dramariasuarez@gmail.com (M.S.A.); martablascoalonso@gmail.com (M.B.A.); dr.narbona@gmail.com (I.N.A.); 2 Department of Surgical Specialties, University of Malaga, 29010 Málaga, Spain

**Keywords:** TAPS, monochorionic pregnancy, intrauterine transfusion, MCA-PSV, fetoscopic laser treatment, expectant management

## Abstract

Twin anemia–polycythemia sequence (TAPS) in monochorionic twin pregnancies is a potentially serious complication caused by unidirectional vascular anastomoses in the placenta, resulting in one anemic donor twin and one polycythemic recipient twin. Diagnosis of this condition is achieved through Doppler ultrasound assessment of the difference between the MoM of the peak systolic velocity of the middle cerebral artery between the twins, establishing the diagnosis with a delta value >0.5 MoM. Management of this situation is individualized and may include intrauterine transfusions, intrauterine laser treatment, and expectant management through ultrasound monitoring of both fetuses to prevent complications. In severe cases, pregnancy termination may be necessary. It is essential that these pregnancies are managed by a multidisciplinary team of professionals, including obstetricians specialized in fetal medicine and neonatologists, to ensure the best possible outcome for both the mother and the fetuses. Early detection and treatment are crucial in the management of pregnancies complicated by twin anemia–polycythemia sequence. The main objective of this article is to conduct a review of the existing literature on the anemia–polycythemia sequence in monochorionic pregnancies, emphasizing the exceptional nature of the presented case due to its spontaneous occurrence, which has a very low prevalence compared to post-laser TAPS cases. It also discusses the different treatment options, highlighting the importance of expectant management and individualization in each case.

## 1. Introduction

Monochorionic (MC) multiple pregnancies have a higher risk of complications compared to dichorionic pregnancies due to the vascular architecture of the placenta and the presence of vascular anastomoses that allow blood flow between the twins. An alteration in the net distribution of this inter-twin flow can lead to various complications, including twin-to-twin transfusion syndrome (TTTS) and twin anemia–polycythemia Sequence (TAPS) [1].

TAPS is a complication of MC pregnancies characterized by a hemoglobin value discrepancy between twins due to the presence of small placental arteriovenous (AV) anastomoses [2], smaller than 1 mm, causing a slow, chronic unidirectional transfusion from the donor twin (anemic) to the recipient twin (polycythemic) [3,4]. Normal MC placentas contain both AV (unidirectional) and arterio-arterial (AA) and veno-venous (VV) (bidirectional) anastomoses, which allow compensatory blood flow between the twins. In most TAPS cases, compensatory AA anastomoses are absent (appearing in only 10–20% of TAPS cases, compared to 25% in TTTS-complicated placentas and 80% in uncomplicated MC placentas), contributing to the occurrence of TAPS due to the lack of compensatory mechanisms [5]. Additionally, there is a slow and unidirectional flow of blood (<15 milliliters/24 h) between the donor and recipient through the AV anastomoses, which contributes to 1% of the fetal blood volume, and causes a progressive hemoglobin (Hb) discordance between the twins. This slow progression allows for hemodynamic compensation, explaining the absence of amniotic fluid volume (AFV) discordance, unlike in TTTS. In TTTS, the anastomoses are larger, and the net transfusion volume is approximately 2% of the fetal blood volume per 24 h, leading to oligohydramnios in the donor and polyhydramnios in the recipient twin [5]. The absence of the oligohydramnios–polyhydramnios sequence is a sine qua non condition for TAPS diagnosis, as it is a pathognomonic sign of TTTS. Thus, TTTS results from a significant blood volume exchange between the donor and recipient, while TAPS involves a hemoglobin concentration difference due to a slow, chronic inter-twin transfusion process [1,2,3]. It is important to note that TAPS, TTTS, and intrauterine growth restriction (IUGR) diagnoses are not mutually exclusive, as TAPS and TTTS can coexist in 15% of cases, and more than 40% of TAPS-affected donor fetuses are also diagnosed with IUGR [5].

There are two types of TAPS according to their cause: spontaneous and post-laser treatment. Spontaneous TAPS complicates 3–5% of MC pregnancies [1,6]. The median gestational age at diagnosis is 23 weeks, and there are cases where the sequence resolves due to spontaneous thrombosis of the AV anastomoses. Post-laser TAPS, a complication of fetoscopic laser surgery for TTTS, occurs in 2–13% of cases [6]. It is due to incomplete laser surgery, leaving some small residual AV anastomoses without compensatory AA anastomoses. This wide complication range is explained by the varying risk based on the number of residual anastomoses left by different laser techniques [1]. The risk decreases with the Solomon technique (2–6%) and increases with selective fetoscopic laser techniques. Post-laser TAPS progresses gradually, typically beginning within the first month after treatment, but can start up to 17 weeks later [5]. In 50% of these cases, the recipient twin becomes the anemic donor, and the former donor becomes the polycythemic recipient [5,7].

## 2. Detailed Case Description

We present a clinical case of a 35-year-old woman with a spontaneous monochorionic–diamniotic twin pregnancy. The pregnancy had a normal course and was monitored at another center until week 26 + 4, when she was referred to our hospital due to uterine contractions and cervical shortening. She was admitted and hospitalized for tocolytic protocol initiation with Atosiban and administration of two doses of 12 mg Betamethasone for lung maturation due to the diagnosis of threatened premature delivery.

**Figure 1:**
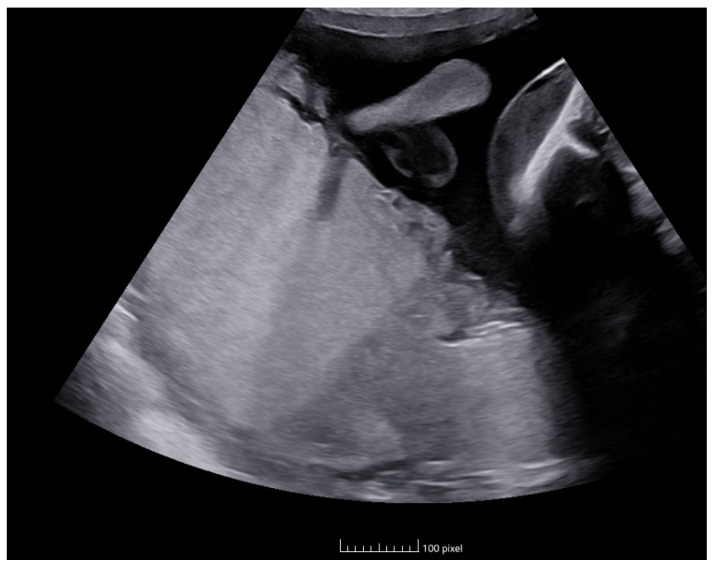
Placenta discordant echogenicity.

During her stay, a control ultrasound at week 27 + 5 revealed the following: the first fetus had an estimated fetal weight (EFW) of 1073 g (27th percentile), a PSV of 55 cm/s (1.53 MoM), a normal UA PI, a visible bladder, and normal AFV; the second fetus had an EFW of 1221 g (74th percentile), a PSV of 34 cm/s (0.94 MoM), a normal UA PI, a visible bladder, and normal AFV. The placenta was located on the posterior wall with discordant echogenicity (Figure 1). Given the findings and the suspicion of TAPS, the case was presented to the multidisciplinary perinatology committee, and it was decided to follow up with ultrasound monitoring twice a week for evaluation.

**Figure 2:**
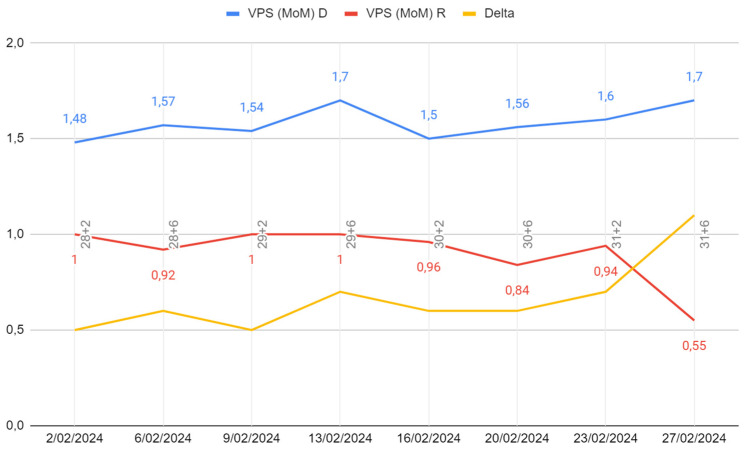
Evolution of PSV and delta value.

In Figure 2, we can observe the evolution of both the PSV and the delta value during subsequent follow-up assessments.

Following the ultrasound values observed at week 31 + 2 (02/27), the first fetus had an MCA-PSV of 24 cm/s (0.55 MoM) with no signs of cardiac compromise, and the second fetus had an MCA-PSV of 70 cm/s (1.7 MoM) with no signs of cardiac compromise, with a delta MCA-PSV value of 1.1 (Stage II).

**Figure 3:**
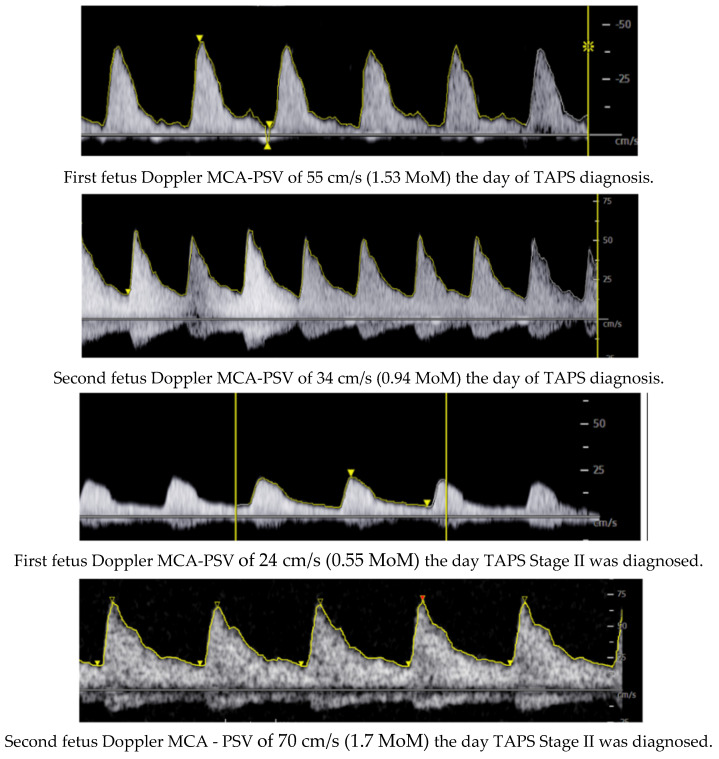
Doppler ultrasonography of evolution of MCA-PSV of the twins.

Due to the progression of the pathology in the last ultrasound check (Figure 3), the case was presented to the obstetric committee, where intrauterine fetal transfusion was dismissed due to the risk of fetal damage during the procedure and the great technical difficulty of accessing the anemic fetus. For this reason, the case was considered as a progressive Stage II, and after ruling out the possibility of intrauterine transfusion, it was decided to schedule an elective cesarean section at 32 weeks for fetal interest, with the first twin in a breech presentation after completing neuroprotection with magnesium sulfate.

The characteristics (weight, fetal Hb value, reticulocytes, and Htc) are summarized in Table 1.

Newborn 1 weighed 1700 g, while Newborn 2 weighed 1450 g. The Hb value of Newborn 1 was 24 g/dL, while that of Newborn 2 was 6 g/dL (a discrepancy of 17.5 g/dL). The reticulocyte percentage of Newborn 1 was 4.6%, while that of Newborn 2 was 16.25% (a difference of 11.65%). Finally, the Hct of Newborn 1 was 69.3%, while that of Newborn 2 was 22%.

Both newborns were admitted to the Intensive Care Unit (ICU). Newborn 1 (recipient) required two doses of surfactant due to respiratory distress and started phototherapy, which was maintained for 7 days. Oxygen therapy was continued for 6 days. The newborn stayed in the ICU for 9 days and then 25 days in the Intermediate Care Unit. Echocardiography and cranial ultrasound during the admission showed normal results. The Hb before discharge was 17.4 g/dL. During follow-up by the Pediatrics Unit at the premature infant follow-up clinic at two months of age, mild conductive hearing loss was detected, and further studies are currently pending.

Newborn 2 (donor) started noninvasive mechanical ventilation (NIMV) with FiO_2_ 21% and phototherapy, received a transfusion of 15 cc/kg of red blood cells, and was treated with iron therapy during the admission. Ventilatory support was maintained for 7 days. The newborn stayed in the ICU for 8 days and then 25 days in the Intermediate Care Unit. Echocardiography and cranial ultrasound during the admission showed normal results. Control blood tests revealed hypothyroidism, which was confirmed at discharge. The Hb before discharge was 10.2 g/dL. As a complication, profound bilateral hearing loss was detected at the premature infant follow-up clinic at two months of age, and auditory brainstem response (ABR) testing is pending.

Our patient has authorized the publication of this clinical case through informed consent.

## 3. Discussion

### 3.1. Prenatal Diagnosis

The prenatal diagnosis of TAPS was traditionally based on the difference in peak systolic velocity (PSV) of the middle cerebral artery (MCA) between both twins. The established cutoff point for predicting TAPS is >1.5 Multiples of the Median (MoM) in the donor fetus (indicative of fetal anemia) and <1 MoM in the recipient fetus (indicative of polycythemia), with a sensitivity of 46%, specificity of 100%, positive predictive value of 100%, and negative predictive value of 70% [2,3,4,8,9].

In 2018, the group led by Tollenaar et al. proposed a new classification and diagnostic system for TAPS based on the delta MCA-PSV value (the difference between the MoM of PSV of the donor and recipient fetuses), establishing a cutoff value for diagnosis of delta > 0.5 MoM. This diagnostic criterion offers a sensitivity of 83%, specificity of 100%, positive predictive value of 100%, and negative predictive value of 88% for predicting TAPS. This change allows us to diagnose TAPS even when the MoM in one of the fetuses is within normal limits. The different stages of TAPS show the progression until it culminates in fetal death; however, this progression may not be linear and can even regress to previous stages. In the first and second columns of the following table, we can observe the prenatal diagnostic criteria for the different stages of TAPS based on classic criteria and the delta value, respectively [3,8,9] (Table 2).

In addition, other ultrasonographic characteristics can support the diagnosis. One of these is discordant placental echogenicity: the placenta corresponding to the donor fetus is thicker and more echogenic compared to that of the recipient fetus. Another described finding is the “Starry sky liver”, where hyperechoic points (corresponding to portal venules) are seen against a background of decreased echogenicity in the liver parenchyma of the recipient twin. Finally, cardiac compromise, Doppler alterations, and fetal hydrops may be observed in the later stages of TAPS [4,5,8,9].

There is no clear consensus on TAPS screening in monochorionic pregnancies. While the Society for Maternal-Fetal Medicine (SMFM) does not recommend routine TAPS screening, it does recommend screening for STFF. The International Society of Ultrasound in Obstetrics and Gynecology (ISUOG) recommends TAPS screening every two weeks starting at 20 weeks’ gestation by measuring the pulsatility index of the umbilical artery [4].

### 3.2. Postnatal Diagnosis

Slow blood transfusion during gestation allows us to observe analytical differences between the twins at birth. The established postnatal diagnostic criteria are an inter-twin Hb difference > 8 g/dL and at least one of the following: reticulocyte ratio > 1.7% (due to increased erythropoiesis resulting from chronic anemia) or the presence of anastomoses < 1 mm on placental examination [1,6,7,9].

This increase in reticulocytes in the donor twin enables differential diagnosis from acute peripartum fetal-to-fetal transfusion (TFF), as in TFF cases, transfusion occurs rapidly, and thus the donor’s reticulocyte count will typically be low. Accurate diagnosis is important because TAPS anemia is euvolemic, whereas anemia in peripartum TFF requires prompt correction of the donor’s hypovolemia [1].

Postnatal staging of TAPS severity is based on the Hb difference between the twins (Table 2). It is important to remember that for both establishing the diagnosis and determining the severity of postnatal TAPS, we rely not on the degree of anemia and polycythemia but on the Hb difference between the two fetuses [3].

We can predict the postnatal stage of TAPS based on ultrasonographic findings during gestation. This prediction will help us decide the best management approach for each case (Table 2) [5].

### 3.3. Management

Once the diagnosis is made, a more extensive Doppler study will be necessary, including the evaluation of blood flow in the umbilical artery and ductus venosus, to establish a stage-based classification of severity. This classification will guide prenatal management, depending on the severity stage, gestational age at diagnosis, the technical feasibility of intrauterine therapy, and parental decisions. Therefore, individualized management of monochorionic pregnancies complicated by TAPS is essential. It is important to note that the anemia–polycythemia sequence is not associated with an increased risk of chromosomal abnormalities, structural anomalies, or other syndromes, so genetic testing is not required in the absence of other markers suggesting chromosomal disorders [4].

The main therapeutic options for TAPS are expectant management through monitoring, termination of the pregnancy, laser photocoagulation, selective feticide, and intrauterine transfusion of the donor fetus, with or without an associated exchange transfusion of the recipient fetus. Although these are fundamental management options, the most significant way to prevent morbidity in TAPS is the modification of fetoscopic laser techniques to reduce the number of residual anastomoses leading to this sequence [4,6].

Expectant management involves performing weekly ultrasounds with a comprehensive Doppler study to determine the severity stage of the pregnancy. If ultrasonographic abnormalities begin to appear, the frequency of these evaluations may be increased. This option is recommended for TAPS diagnosed during the first trimester or early second trimester, as these cases tend to remain stable or even resolve spontaneously. In Stages II or lower, expectant management may be attempted with the goal of completing the pregnancy at a late preterm stage. Conversely, if TAPS continues to progress, alternative treatments may be considered [5].

Intrauterine transfusion of the anemic twin is not a causal treatment but can provide a temporary solution and extend the time until birth, reducing risks associated with extreme prematurity. This therapy can be performed via intravascular or intraperitoneal routes. A combined procedure of donor transfusion and recipient exchange transfusion could reduce this risk. This type of treatment is not recommended for rapidly progressing TAPS cases, as there is an observed increased risk of rapid blood transfer to the recipient twin, worsening their clinical condition. If progression to a higher stage occurs after an initial transfusion or if anemia rapidly reappears in the donor twin, alternative therapeutic approaches should be considered [5].

Fetoscopic laser treatment is the only option with potential curative effects for TAPS. The complexity of this technique is high due to the difficulty in accurately locating the vascular equator of the placenta. Both the size and number of anastomoses are low, making it essential to plan the surgery in advance. The echogenicity difference of the placenta, fetal size, and the location of the umbilical cord insertions can help locate the vascular equator [6].

In cases where it is determined that the entire vascular equator cannot be visualized during the intervention, selective reduction of one of the twins might be considered a radical option [5].

In a large cohort study conducted by Tollenaar’s group, including both post-laser TAPS and spontaneous TAPS cases from 17 different centers between 2014 and 2019, a highly heterogeneous management approach was observed due to the need for individualized treatment in each case [10].

In the absence of clear evidence regarding the optimal management of TAPS, Tollenaar et al. suggest that decisions on management should be individualized, considering various factors such as the severity stage, gestational age, and technical feasibility of performing intrauterine treatments [1].

While emphasizing the importance of individualized treatment based on each patient’s specific characteristics, Tollenaar et al. propose this management algorithm based on expert opinion [1] (Table 3).

According to the staging, the management will differ:

In pregnancies with TAPS Stage I, expectant management with weekly Doppler studies will be considered to monitor possible progression to a higher stage. In the absence of progression, the pregnancy may be concluded between 34 + 0 and 37 + 0 weeks.

If the condition is Stage II, pulmonary maturation with corticosteroids is recommended. In pregnancies <32 + 0 weeks, expectant management with Doppler studies twice a week may be opted for. If the condition remains stable, the pregnancy should be concluded between 32 + 0 and 34 + 0 weeks. If there is progression to a more severe stage in pregnancies <32 weeks, management will be based on the severity stage to which it progresses. In pregnancies with TAPS Stage III, there is no clear consensus on the most appropriate management, thus requiring individualized decision-making [1].

For spontaneous TAPS cases before 28 weeks of gestation, laser ablation is considered the best option. Alternatively, intrauterine transfusion could be considered. After treatment, weekly ultrasound monitoring will be necessary until resolution of the sequence. For TAPS cases between 28 and 32 weeks of gestation, both transfusion and fetal laser therapy are acceptable options. After treatment, weekly ultrasound monitoring is required until resolution. If anemia–polycythemia persists in Stage II, III, or IV after 32 weeks of gestation, termination of pregnancy is indicated [1].

The best strategy for preventing TAPS would be to modify the laser ablation technique by using the Solomon technique, which reduces the risk of TAPS from 2–13% following STFF therapy to 2–6% [1,11].

### 3.4. Prognosis

Up to 16% of known cases of TAPS have shown spontaneous resolution, often due to spontaneous thrombosis of the AV anastomoses [2].

Spontaneous cases generally have a favorable prognosis and may be managed expectantly. However, cases occurring after laser treatment for STFF tend to be more aggressive and often require treatment [2].

The outcomes for twin pregnancies affected by TAPS are highly variable. Severe TAPS can result in the death of one or both fetuses. Conversely, mild TAPS frequently results in two healthy neonates, although discrepancies in Hb levels may necessitate transfusion and exchange transfusion procedures for the twins. Recipients may experience necrosis and ischemia in various locations due to increased blood viscosity. Donor fetuses, due to the transfusion of albumin in addition to Hb, may suffer decreased oncotic pressure, potentially leading to fetal hydrops. The primary neonatal morbidities are anemia and polycythemia; however, severe brain damage has been reported in advanced stages. Recently, long-term complications such as bilateral sensorineural hearing loss, spastic paralysis, and cognitive delay have also been described [1,11].

## 4. Conclusions

Spontaneous TAPS is an uncommon complication of monochorionic twin pregnancies caused by the presence of unidirectional AV anastomoses, leading to chronic inter-twin transfusion and resulting in Hb discrepancies between an anemic twin and a polycythemic one. The classical diagnostic criterion was based on the VPS-ACM values of each twin separately. Currently, the delta value (>0.5 MoM), which is the difference between the VPS-ACM values of both twins, is used as it shows a better correlation with postnatal outcomes. Screening for TAPS in monochorionic twin pregnancies is recommended with Doppler monitoring every 2 weeks, starting from 20 weeks of gestation. Prenatal management depends on the severity stage, gestational age, and technical feasibility of intrauterine therapies.

The prognosis is variable, with the presence of TAPS increasing the risk of developmental abnormalities. Notably, the prognosis for spontaneous TAPS is generally better than for TAPS following laser treatment.

Therefore, given the limited evidence on the outcomes and optimal management of TAPS, treatment should be individualized for each case and discussed with the parents.

## Figures and Tables

**Table 1:** Fetal characteristics.

	Recipient	Donor
date of birth	29 February 2024	29 February 2024
weight	1700 gr	1450gr
Apgar	8/9	6/9
pH	7.16	7.16
hemoglobin	24 g/dL	6.5 g/dL
hematocrit	69.3%	22%
reticulocyte count	272 cells/µL	265 cells/µL
reticulocyte percentage	4.6%	16.2%

**Table 2:** Stages of TAPS based on classic diagnostic criteria, delta value, and discordance of postnatal hemoglobin levels.

Stage	Classical Classification Criteria	Delta Value-Based Classification Criteria
Stage I	ACM-PVS donor > 1.5 MoM, recipient < 1 MoM.	Delta ACM-PVS > 0.5 MoM.
Stage II	ACM-PVS donor > 1.7 MoM, recipient < 0.8 MoM.	Delta ACM-PVS > 0.7 MoM.
Stage III	Stage I or II associated with donor cardiac compromise *.	Stage I or II associated with donor cardiac compromise.
Stage IV	Fetal hydrops donor.	Fetal hydrops donor.
Stage V	Death	Death

* Flow alteration: absence of diastole or reverse flow in the umbilical artery, pulsatile flow in the umbilical vein, and/or increased pulsatile flow index/reverse flow in the ductus venosus; * ACM-PVS: peak systolic velocity of the middle cerebral artery; MoM: Multiples of the Median.

**Table 3:** Proposed antenatal management of TAPS [1].

<28 weeks	Stage 1	Expectant management
	Stage ≥ 2	Laser treatment
28–32 weeks	Stage 1	Expectant management
	Stage 2 WITHOUT progression	Laser treatment
	Stage 2 WITH progression	Intrauterine transfusion
	Stage > 3	Intrauterine transfusion
>32 weeks	Stage 1	Expectant management
	Stage 2 WITHOUT progression	Expectant management
	Stage 2 WITH progression	Terminate pregnancy
	Stage > 3	Terminate pregnancy

## Data Availability

The datasets used in the preparation of this article are available upon request from the corresponding author.
